# Plasmonic Nanomaterial-Based Optical Biosensing Platforms for Virus Detection

**DOI:** 10.3390/s17102332

**Published:** 2017-10-13

**Authors:** Jaewook Lee, Kenshin Takemura, Enoch Y. Park

**Affiliations:** 1 Research Institute of Green Science and Technology, Shizuoka University, 836 Ohya Suruga-ku, Shizuoka 422-8529, Japan; lee.jaewook@shizuoka.ac.jp; 2 Department of Applied Biological Chemistry, College of Agriculture, Graduate School of Integrated Science & Technology, Shizuoka University, 836 Ohya Suruga-ku, Shizuoka 422-8529, Japan; takemura.kenshin.16@shizuoka.ac.jp

**Keywords:** plasmonic nanomaterial, metal nanoparticle, nanoparticles, decorated carbon nanomaterial, optical biosensing system, virus detection

## Abstract

Plasmonic nanomaterials (P-NM) are receiving attention due to their excellent properties, which include surface-enhanced Raman scattering (SERS), localized surface plasmon resonance (LSPR) effects, plasmonic resonance energy transfer (PRET), and magneto optical (MO) effects. To obtain such plasmonic properties, many nanomaterials have been developed, including metal nanoparticles (MNP), bimetallic nanoparticles (bMNP), MNP-decorated carbon nanotubes, (MNP-CNT), and MNP-modified graphene (MNP-GRP). These P-NMs may eventually be applied to optical biosensing systems due to their unique properties. Here, probe biomolecules, such as antibodies (Ab), probe DNA, and probe aptamers, were modified on the surface of plasmonic materials by chemical conjugation and thiol chemistry. The optical property change in the plasmonic nanomaterials was monitored based on the interaction between the probe biomolecules and target virus. After bioconjugation, several optical properties, including fluorescence, plasmonic absorbance, and diffraction angle, were changed to detect the target biomolecules. This review describes several P-NMs as potential candidates of optical sensing platforms and introduces various applications in the optical biosensing field.

## 1. Introduction

Plasmon has an interesting property that occurs as an electron cloud on the surface of metallic nanomaterials [1,2,3]. When metallic nanoparticles (MNP) interact with incident light, the electron cloud in the MNP oscillates, and in this case, the collective excitation of electrons is regarded as plasmon [1,4]. Recently, the plasmonic effect induced by pi electrons has been discovered in carbon nanomaterials, such as graphene (GRP) and carbon nanotubes (CNT) [5,6,7,8]. In some cases, plasmonic properties can be tuned by interactions between plasmonic nanomaterials (P-NM) and shape control, thus controlling their optical properties to exhibit various colors [9,10,11,12,13].

Numerous P-NMs have been introduced, including MNP, such as gold, silver, platinum, CNT, and GRP. To achieve an enhanced plasmonic effect, hybrid plasmonic nanomaterials have been developed, such as bimetallic NP (bMNP), core/shell MNP, MNP-decorated CNT (MNP-CNT), and MNP-modified GRP (MNP-GRP) [14,15,16,17]. To prepare these P-NMs, various synthetic strategies have been suggested, such as chemical reduction processes, electrochemical deposition, microwave-assisted synthesis processes, photo-induced decoration processes, and environmentally friendly processes [18,19,20,21]. In addition, such P-NMs possess excellent optical properties, such as surface enhanced Raman scattering (SERS), plasmonic resonance energy transfer (PRET), enhanced catalytic properties, a localized surface plasmon resonance (LSPR) effect, and the magneto optical (MO) effect [22,23,24,25,26]. 

Because P-NMs have such specialized optical properties, they are reported to have many potential applications including energy devices, nano optics, sensors, and nanobiomedicine [27,28,29,30,31,32,33]. Among these, many researchers are investigating development of highly sensitive and selective biosensing systems to address public concerns and health care issues [34,35,36]. In addition, P-NMs have improved the specificity of on-site detection and point-of-care diagnosis platforms. Attempts to monitor several infectious diseases have been made using P-NM-based sensing systems, including the influenza virus, norovirus, and tuberculosis (TB) [37,38,39]. Viral DNA and RNA have also been detected using P-NMs [30,40]. 

In this review, we introduce various P-NMs, such as MNPs, bMNPs, MNP-CNT, and MNP-GRP, as candidates for optical sensing platforms. We also describe several P-NM-based optical biosensing systems that are demonstrated via SERS, PRET, LSPR, and colorimetry, including viral DNA and RNA detection. 

## 2. Various Functions of Plasmonic Nanomaterials (P-NMs)

P-NMs can be classified into in two categories: noble metal-based MNP and MNP-carbon nanomaterials including MNP-CNT and MNP-GRP. Both P-NMs have excellent plasmonic and optical properties. Among of these, noble metals, such as Au, Ag, and Pt-based MNPs, are well-established and widely applied in various fields [41,42] due to their stable plasmonic properties and particle stability [43]. In addition, MNP’s plasmonic property can be easily tuned and controlled through particle structure or the distance between each MNP. On the other hands, tunable infra-red (IR) plasmonic property of GRP also has been interested due to excellent optical properties such as enhancement of light and matter interaction and integration of mid-IR photonics [44].

**Figure 1:**
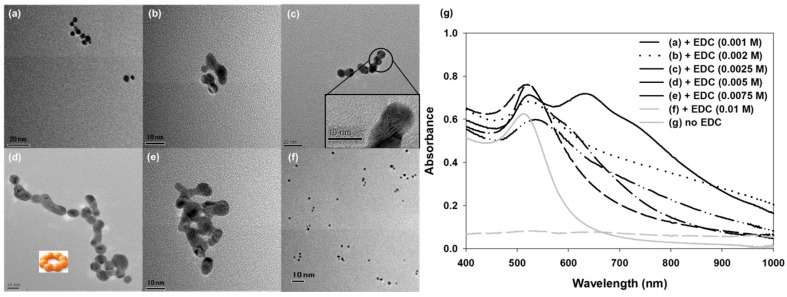
Various structure of Au-nanoparticles (NPs) and their plasmonic property. (**a**–**f**) Structural differences in AuNPs using TEM and (**g**) plasmonic absorbance measurement after adding varying concentrations of EDC. Reproduced from Ref. [46] with permission from the Royal Society of Chemistry.

Oh et al. tuned the plasmonic property of AgNP by controlling its particle size [45]. In this case, the size was changed by the seed-mediated growth method, and as the Ag ion concentration increased, large AgNPs were produced. In addition, the plasmonic absorbance of AgNP was redshifted, so the solution color changed from yellow to red. Lee et al. tuned the plasmonic property of AuNP by controlling the AuNP assembly (Figure 1) [46]. Typically, the plasmonic absorbance of spherical AuNP is measured to be approximately 525 nm. However, in this study, the authors synthesized AuNP chains using *N*-(3-Dimethylaminopropyl)-*N’*-ethylcarbodiimide (EDC) and as the EDC concentration increased, the AuNP chain was elongated, changing its structure (Figure 1a–f). Thus, the absorbance tendency of the AuNP chain changed and was redshifted. This phenomenon occurs by the plasmonic coupling effect between AuNPs that are in close contact, and this effect may have induced the secondary plasmonic band that was observed at approximately 625 nm (Figure 1g).

**Figure 2:**
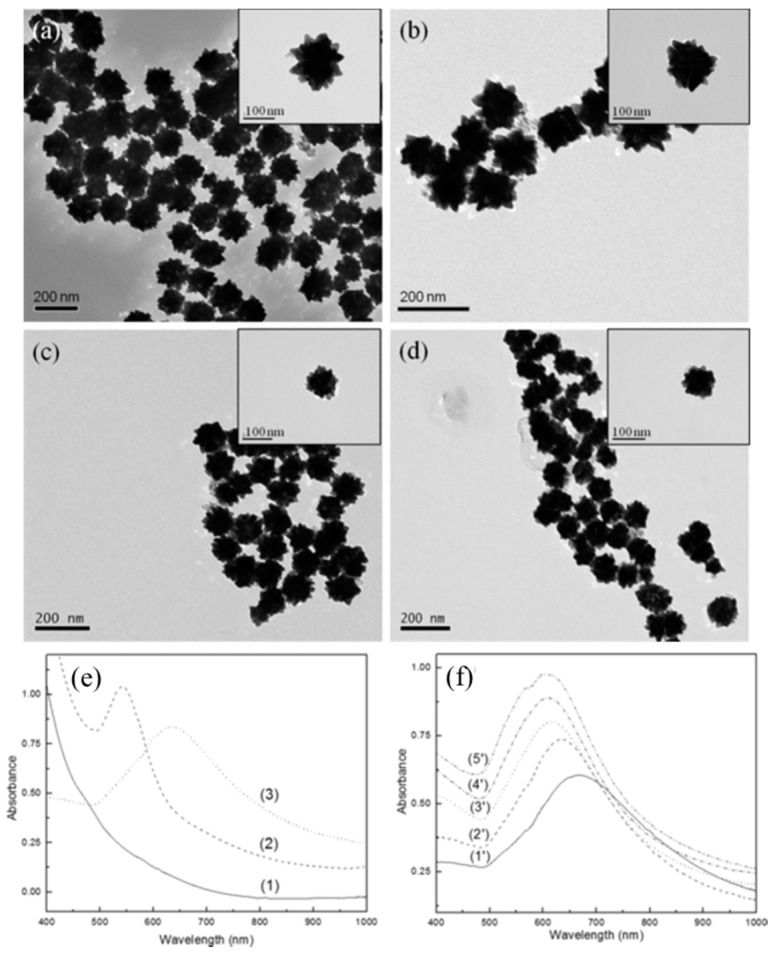
Urchin-shaped iron oxide-AuNP core/shell structure. (**a**–**d**) TEM images of iron oxide-AuNP core/shell structure; (**e**) UV/Vis absorbance spectra of (1) iron oxide, (2) spherical iron oxide@gold core/shell and (3) urchin type of iron oxide@gold core/shell and (**f**) plasmonic absorbance spectra of urchin-shaped structure depending on the concentration of seeds for assembly (1’) 4.5 nM, (2’) 8.9 nM, (3’) 13.2 nM, (4’) 17.5 nM and (5’) 21.7 nM. Copyright Wiley and Sons. Reproduced with permission [47].

To improve P-NM functionality and obtain a synergic effect, many core/shell structures have been developed. Zhou et al. reported an iron oxide AuNP core/shell particle structure that was urchin-shaped rather than spherical (Figure 2a–d) [47]. Thus, it could possess multi-functional properties including magnetic and plasmonic properties. In addition, this particle exhibited attractive optical properties due to the structurally induced plasmonic effect. This spiky structure could generate a plasmonic hotspot, and its electromagnetic field could be more enhanced than in the spherical case. Thus, this structure could possess improved SERS and catalytic activities [1]. In this case, the broadened absorbance spectrum of urchin-shaped structure was measured from 500 nm to 800 nm (Figure 2e). This urchin-shaped particle was prepared by the seed mediated synthesis method, and its plasmonic property was tuned depending on the concentration of seed material (Figure 2f). In another study, an Au-Ag core/shell structure was introduced to improve and tune the plasmonic property [48]. In this case, two plasmonic absorbance peaks were obtained, one approximately 424 nm and one with a broad peak between 538 nm and 560 nm. Using the plasmonic property of Au-Ag P-NM, they developed an optical sensing platform to detect the Zika virus. In other study, P-NM played a role as MO active substrate to detect a single molecule via light control [49,50]. In this case, single organic molecule has been detected under the strong external magnetic field with Au/Ag bimetallic hybrid structure that was used as MO active material [50]. 

**Figure 3:**
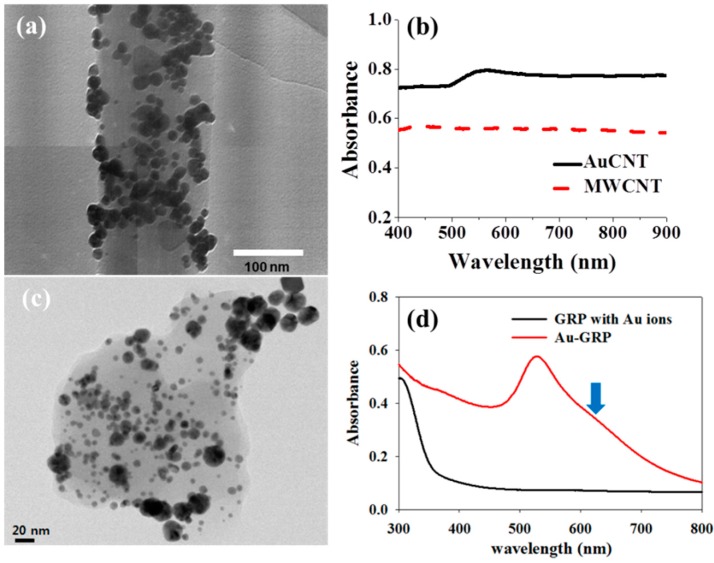
Structure of Au-CNT and Au-GRP. TEM images of (**a**) Au-CNT; (**b**) plasmonic absorbance of Au-CNT; (**c**) Au-GRP and (**d**) UV/Vis spectrum of Au-GRP as hybrid P-NM structures. (**a**,**b**) Copyright Elsevier B.V. Reproduced with permission [51] and (**c**,**d**) Reprinted with permission from [52], Copyright 2014, American Chemical Society.

Recently, MNP decorated carbon nanomaterial (MNP-CNM) has been considered as a potential P-NM due to its excellent synergic properties, such as SERS, PRET and MO. Because CNM possesses pi electrons on its surface, plasmonic coupling between MNP and the CNM surface could occur. Thus, a strong and longitudinal plasmonic effect can be induced. For example, AuNP-CNMs were prepared as P-NM by Lee and colleagues, and in this study, AuNP-CNT and AuNP-GRP were synthesized through simple two steps without harsh conditions (Figure 3) [51,52]. In this case, Au ions and CNMs were sonicated in deionized water, then mild reducing agent was added to the mixture, and it was stirred for 3 h to obtain AuNP-CNT or AuNP-GRP. After preparation of hybrid material, its morphology was observed by TEM and in this case, a lot of Au NPs were well attached and distributed on the surface of CNT or GRP (Figure 3a,c). The plasmonic property of MNP-CNM was also characterized by UV/vis spectroscopy and as a result, plasmonic absorbance of MNP-CNM was clearly measured that was shown in Figure 3b,d. Using these hybrid nanomaterials, a PRET-based fluoro-immunoassay detected an infectious virus. In addition, AgNP-CNT was fabricated to enhance the property [53]. This hybrid structure served as a SERS substrate, and its sensitivity to the R6G molecule was enhanced by ~10^10^. Other type of core/shell was also developed that was Au@cage (Au/Ag bimetallic cage) and this Au@cage structure was used for SERS active material to detect a malaria virus DNA [54]. It also showed highly sensitive detection performance and its LOD was about 100 attomole.

Various P-NMs have been developed and investigated to use their specialized plasmonic and optical properties. They can be tuned, and synergic effects can be obtained through various strategies, such as assembly, core/shell, and hybrid structure with CNM.

## 3. Application of P-NM-Based Biosensing Systems

### 3.1. LSPR and PRET-Based Sensing Platform

P-NMs can be applied in various fields, such as nano optics, drug delivery systems (DDS), contrast agents, and sensing systems. Eventually, these plasmonic materials may play key roles in biosensing platforms. By using P-NMs, researchers have developed several types of biosensing systems, including SERS and LSPR-based optical sensing platforms, PRET-based fluoro-immunoassays, and colorimetry-based detection systems. These P-NM-based biosensing platforms can detect viral antigens and viral DNA or RNA, thus this system could be applied universally. 

**Figure 4:**
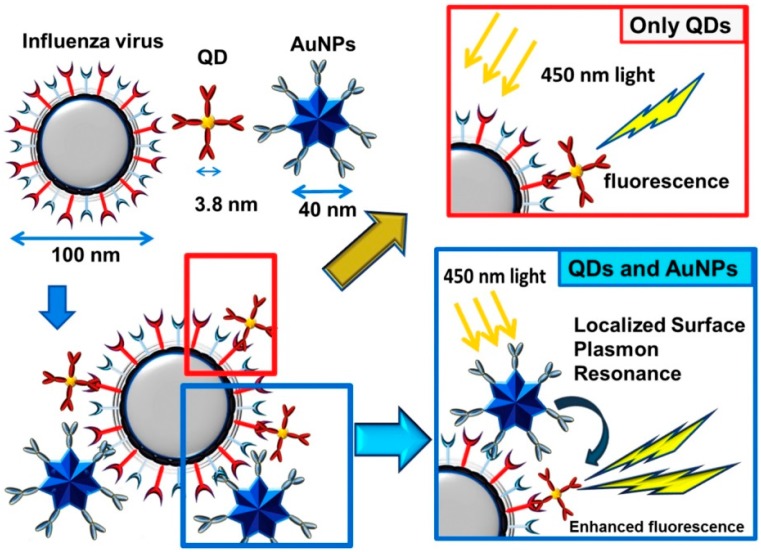
Schematic illustration of the localized surface plasmon resonance (LSPR)-based detection mechanism of biosensing for the influenza virus. Copyright Elsevier B.V. Reproduced with permission [55].

Takemura and colleagues developed LSPR based-sensing systems to detect an influenza virus, and non-spherical AuNPs were used as plasmonic materials (Figure 4) [55]. 

Fluorescent quantum dot (QD) was used to monitor the viral antigen. If QD is located near the P-NM, its fluorescence intensity can be enhanced by the LSPR-based PRET effect [56,57,58]. Therefore, if the target virus existed in the antibody (Ab) modified P-NM/QD system, key and lock interaction would occur between the target virus and P-NM/QD. As a result, the fluorescence intensity of QD could be enhanced by energy transfer in the virus/P-NM/QD structure. Depending on the concentration of viral antigen, the fluorescent enhancement factor was affected, and as the amount of the virus increased, the QD fluorescence gradually increased. In this study, the limit of detection (LOD) for the influenza virus was estimated at 0.03 pg/mL in deionized water and 0.4 pg/mL in a serum matrix. In addition, clinically isolated influenza virus was detected with 10 PFU/mL detection limitations. Moreover, this system was highly selective against other biomolecules. In a different study, Zika virus RNA was monitored using Au-Ag core/shell P-NM and QD [48]. The target RNA was detected by a similar approach to the influenza method. The molecular beacon was modified on the QD surface to capture the target RNA. When Zika virus RNA was placed in this system, hybridization occurred, resulting in enhanced fluorescence intensity. In this case, the LOD was 2.4 copies/mL, with extremely high selectivity.

**Figure 5:**
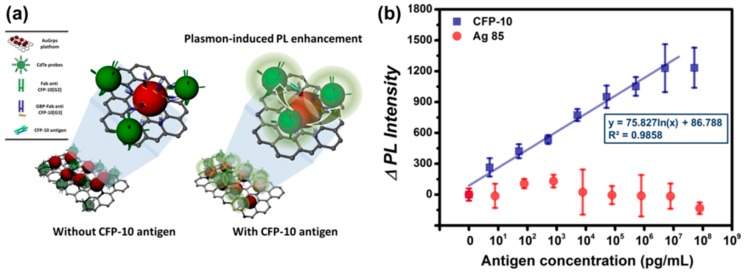
TB antigen detection using Au- Graphene (GRP)-assisted fluoro-immunoassay. (**a**) Au-GRP-assisted fluoro-immunoassay process and (**b**) detection behavior of the plasmonic resonance energy transfer (PRET)-based biosensing system for TB monitoring. Reprinted with permission from [52]. Copyright 2014, American Chemical Society.

MNP-GRP or MNP-CNT can also be used as LSPR substrate for PRET-based sensing systems to monitor the viral antigens. Lee et al. detected infectious TB antigen by using TB Ab-modified Au-GRP and QD through PRET-based fluoro-immunoassay (Figure 5a) [52]. In this study, a sandwich structure was formed with Au-GRP and QD by the TB antigen, i.e., CFP-10 and fluorescence enhancement of QD was measured by the antigen concentration. In Figure 5b, the sensing performance is depicted, and as the target antigen concentration increased, the fluorescence intensity linearly increased. The LOD was estimated at 4.5 pg/mL. Moreover, other TB antigens, such as Antigen 85, were not monitored, thus it showed excellent selectivity. Au-CNT can also be a P-NM for fluoro-immunoassay, and the influenza virus was monitored by a similar strategy with an Au-GRP sensing mechanism [51]. Here, Au-CNT/QD was assembled as a sandwich structure by the influenza virus, resulting in enhanced fluorescence with each virus concentration. It was highly sensitive with 0.1 pg/mL, and a clinically isolated influenza virus was also detected with a 50 PFU/mL detection limitation.

Recently, other type of plasmon resonance mode also could be applied for biosensors such as Fano resonance. According to several literatures, the sensitivity of Fano resonance based detection system could be higher than that of conventional SPR based biosensing system [59,60,61]. 

### 3.2. SERS-Based Sensing Platform

**Figure 6:**
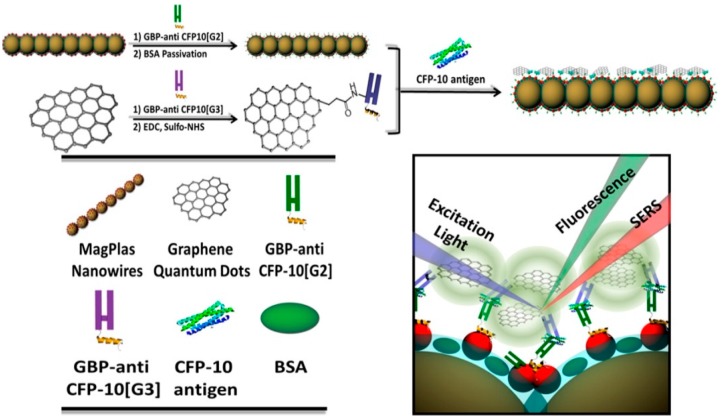
Illustration of the detection process for surface-enhanced Raman scattering (SERS)-based TB biosensing. Reprinted with permission from [66]. Copyright 2015, American Chemical Society.

The SERS effect on P-NMs has a remarkable optical property, and scientists have used it to develop a biosensing platform [62,63,64,65]. Zou et al. fabricated the SERS platform using an iron oxide-Au magnetoplasmonic NP (IO-Au) core/shell P-NM (Figure 6) [66]. Interestingly, IO-Au could be aligned and deposited on the substrate surface through an external magnetic field due to its magnetic property. Therefore, the SERS platform could be easily fabricated without expensive equipment or facilities. In their study, TB antigens were monitored using an IO-Au-based SERS platform and GRP QD. To detect a TB antigen, the TB Ab was modified on the IO-Au and GRP QD surface, and the Raman intensity of the GRP QD was monitored based on the TB antigen concentrations. Here, the linear calibration curve was obtained, and the Raman intensity of the GRP QD increased with an increase in TB antigen. The LOD was calculated at 0.0511 pg/mL. In another study, a reusable AgNP-GRP-based SERS platform was fabricated to detect methylated DNA [67]. The target DNA was captured by Ab-modified AuNPs, and this complex was attached to the surface of the AgNP-GRP-based sensing platform. The Raman intensity changed with the level of DNA methylation, and large increases in the Raman intensity were measured at higher methylation levels. In addition, as the DNA concentration increased, the Raman signal also increased. In this case, the LOD was estimated at 1.8 pg/mL, and the SERS platform was reused after washing it with deionized water and ethanol.

### 3.3. Enzyme Like Activity-Based Colorimetry Sensing Platform

**Figure 7:**
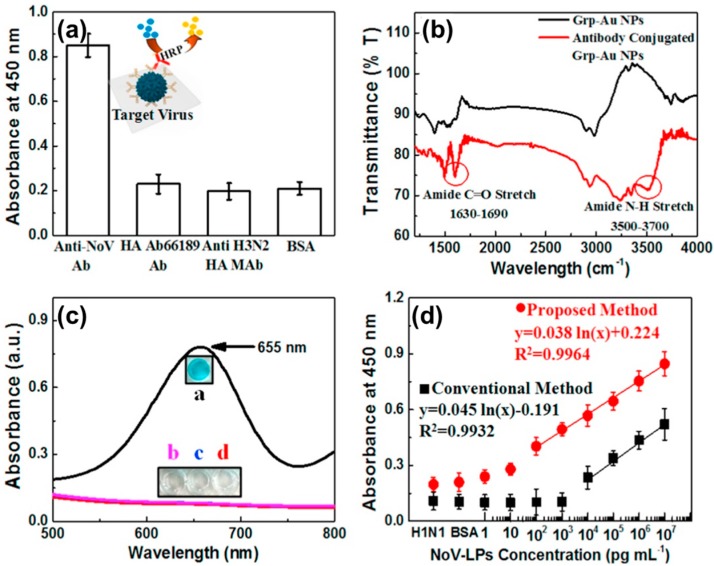
P-NM-based plasmonic application on colorimetry sensing platform. (**a**) Selectivity test with various viruses; (**b**) Confirmation of Ab conjugation with Au-GRP; (**c**) Catalytic activity test and (**d**) Biosensing demonstration with various concentrations of Norovirus-like particles (No-VLPs). Copyright Elsevier B.V. Reproduced with permission [69].

Such P-NM-based plasmonic structures could be applied to a colorimetry sensing platform due to the catalytic properties from free electrons [68]. This means that P-NM possesses enzyme activities, and this material-based sensing system can be an alternative to the enzyme-linked immunosorbent assay (ELISA) system [68]. For example, Rahin and colleagues detected Norovirus-like particles (No-VLPs) using Au-GRP as an enzyme-like catalyst (Figure 7) [69]. First, a specificity test was confirmed on various viruses and a color change was only observed in No-VLP (Figure 7a). Ab conjugation with Au-GRP was confirmed by FT-IR analysis (Figure 7b). In this case, Au-GRP induced the oxidation of the peroxidase substrate 3,3,5,5-tetramethylbenzidine (TMB) with H_2_O_2_, resulting in the appearance of a blue color in the aqueous solution (Figure 7c). Through an improved colorimetry sensing response, the LOD for the No-VLPs in this system was improved to 92.7 pg/mL, which is 112 times higher than that of conventional ELISA (Figure 7d).

In a similar process, the influenza virus was monitored by Au-CNT-induced catalytic activity [70]. This case was highly sensitive at a 3.4 PFU/mL detection limitation, which is 385 times that of the ELISA detection limitation and 500 times that of commercial immunochromatography kits.

## 4. Conclusions

In recent years, many P-NMs have been developed for various applications, and their plasmonic properties have been improved and enhanced to achieve effective performance. Several P-NMs, such as MNPs, bMNPs, MNP-CNT, and MNP-GRP, were applied for novel biosensing platforms, such as the LSPR-based detection system, the SERS substrate, the PRET-induced sensing system, and colorimetry. Moreover, P-NM-based biosensing systems can detect antigens, DNA, and RNA in various diseases with excellent sensitivity and selectivity. Therefore, P-NM-based detection platforms can be widely used as highly accurate biosensing devices for public health. 

## Abbreviation

Antibodies (Ab); Bimetallic nanoparticles (bMNP); Carbon nanotubes (CNT); enzyme-linked immunosorbent assay (ELISA); Graphene (GRP); Infra-red (IR); Iron oxide-Au magnetoplasmonic NP (IO-Au); Limit of detection (LOD); Localized surface plasmon resonance (LSPR); Magneto optical (MO); Metal nanoparticles (MNP); MNP decorated carbon nanomaterial (MNP-CNM); MNP-decorated CNT (MNP-CNT); MNP-modified GRP (MNP-GRP); *N*-(3-Dimethylaminopropyl)-*N′*-ethylcarbodiimide (EDC); Norovirus-like particles (No-VLPs); Plasmonic nanomaterials (P-NM); Plasmonic resonance energy transfer (PRET); Quantum dot (QD); Surface-enhanced Raman scattering (SERS); Tuberculosis (TB); 3,3,5,5-tetramethylbenzidine (TMB).
